# Chromatin changes in response to drought, salinity, heat, and cold stresses in plants

**DOI:** 10.3389/fpls.2015.00114

**Published:** 2015-03-02

**Authors:** Jong-Myong Kim, Taku Sasaki, Minoru Ueda, Kaori Sako, Motoaki Seki

**Affiliations:** ^1^Plant Genomic Network Research Team, RIKEN Center for Sustainable Resource Science, Yokohama, Japan; ^2^Core Research for Evolutional Science and Technology, Japan Science and Technology, Kawaguchi, Japan; ^3^Kihara Institute for Biological Research, Yokohama City University, Yokohama, Japan

**Keywords:** abiotic stress response, DNA methylation, epigenetic regulation, histone modification, stress memory

## Abstract

Chromatin regulation is essential to regulate genes and genome activities. In plants, the alteration of histone modification and DNA methylation are coordinated with changes in the expression of stress-responsive genes to adapt to environmental changes. Several chromatin regulators have been shown to be involved in the regulation of stress-responsive gene networks under abiotic stress conditions. Specific histone modification sites and the histone modifiers that regulate key stress-responsive genes have been identified by genetic and biochemical approaches, revealing the importance of chromatin regulation in plant stress responses. Recent studies have also suggested that histone modification plays an important role in plant stress memory. In this review, we summarize recent progress on the regulation and alteration of histone modification (acetylation, methylation, phosphorylation, and SUMOylation) in response to the abiotic stresses, drought, high-salinity, heat, and cold in plants.

## INTRODUCTION

Past decades, innovation of DNA sequencing technology has brought an evolution of genome biology. Genome projects of many organisms have revealed complexity of genome structure. However, DNA sequence information is not enough to understand the mysterious life phenomenon and how genome is organized. For this, other factors, e.g., chromatin regulation mediated by histone modification and DNA methylation, and RNA-mediated regulation are also involved. Study on such regulation other than genetic information is called as “epigenetics.”

Epigenetic regulation is an important mechanism that is involved in a wide range of biological phenomena, such as genome stability, developmental programming, gene expression, and diseases like cancer through chromatin regulation, small RNA-mediated regulation, and so on (Grewal and Jia, 2007; Feng et al., 2010; Kanherkar et al., 2014). Chromatin regulation mediated by histone modifications and DNA methylation, can be dynamically and statically changed to maintain gene and genome activities (Wolffe, 1998; Zhang and Reinberg, 2001; Kurdistani et al., 2004; Pokholok et al., 2005). Plants perceive stimuli from the surrounding environment and possess sophisticated regulatory networks of genes that can control the accumulation of metabolites, which allows the plants to survive and adapt to environmental changes (Shinozaki and Yamaguchi-Shinozaki, 2007; Urano et al., 2010). Recent studies have reported that histone modifications, such as H3K4me3, H3K9ac, H3K9me2, H3K23ac, H3K27ac, H3K27me3, and H4ac, along with DNA methylation can be correlated with gene expression in response to abiotic stresses, such as water deficit, high-salinity, and temperature shifts (Kim et al., 2008; Luo et al., 2012a). Some histone modifications change rapidly in response to environmental changes, while others change gradually along with changes in gene expression to control physiological homeostasis and development under environmental stresses (Kim et al., 2008, 2012). It still remains unclear which comes first transcriptional changes or chromatin changes, and how they are linked. In this paper, we review recent studies of chromatin regulation in plants, focusing mainly on histone modification and DNA methylation in response to abiotic stresses.

## HISTONE PROTEINS AND MODIFIERS IN PLANTS

A basic core histone octamer for nucleosomes is composed of histones H2A, H2B, H3, and H4 (Arya and Schlick, 2009; Zhou et al., 2013). Histone variants, such as H2A.Z and CENH3, function in precise and specific regulation of gene activity and genome structure (Deal et al., 2007; Zhang et al., 2008; Yan et al., 2010; Coleman-Derr and Zilberman, 2012).

The N-terminal region of histones is called the histone tail. Histones are enriched with basic amino acid residues such as lysine and arginine. The basic residues in histone tails are covalently modified by methylation, acetylation, phosphorylation, and ubiquitination, and these modifications alter the activity of the genes that are wrapped around the core histones. Histone modification can have different effects depending on which residue is modified and the type of modification. The current understanding of the effect of histone modifications is based mainly on research in yeast. In yeast, mutants in which the modified residue in the histone tail is replaced by a different residue have been used to determine the effects of histone modification (Rundlett et al., 1998; Suka et al., 2001).

In *Arabidopsis*, histone modification sites were identified by mass-spectrometry and biochemical assays (Earley et al., 2007; Zhang et al., 2007). The results of these studies suggested that post-translational modification sites of histones in *Arabidopsis* were highly conserved with other eukaryotes. Generally, the acetylation of lysine residues in H3 and H4 N-tails neutralizes the positive charge of the histone tails, which decreases their affinity for DNA and alters the accessibility of transcription factors to the template DNA strand. As a result, histone acetylation tends to induce gene activation (Kuo et al., 1996; Zhang et al., 1998; Shahbazian and Grunstein, 2007). Conversely, the removal of histone acetylation can lead to gene repression and silencing (Kadosh and Struhl, 1998; Rundlett et al., 1998; Chen et al., 2010b; To et al., 2011a). The effects of histone methylation events vary depending on the site of the modification. Although changes in histone modifications can be correlated with gene activity, the molecular mechanisms through which the chemical modifications influence chromosomal structure and the accessibility of transcription factors are still not fully understood. For example, tri-methylation of the fourth lysine of H3 (H3K4me3) is an active mark for gene expression, and tri-methylation in the twenty-seventh lysine of H3 (H3K27me3) is a repressive mark of facultative heterochromatin (Cao et al., 2002; Finnegan and Dennis, 2007; Doyle and Amasino, 2009). These relationships between the alteration of histone modifications and gene activity are highly conserved from yeast to human, and also in plants.

Histone modifiers are also well conserved in angiosperms. For example, the major histone modifiers histone acetyltransferases (HATs), histone deacetylases (HDACs), histone methyltransferases (HMTs), and histone demethylases (HDMs) have been isolated or identified in several plants, including *Arabidopsis*, tomato, rice, barley, grapevine, *Brassica*, and *Brachypodium*. These histone modifiers have been phylogenetically analyzed and classified (Pandey et al., 2002; Chen et al., 2010b; Papaefthimiou et al., 2010; Pontvianne et al., 2010; Aquea et al., 2011; Huang et al., 2011; Aiese-Cigliano et al., 2013), revealing that histone modifiers, such as the HATs, are conserved in plants (Aiese-Cigliano et al., 2013). However, the molecular functions of many of these modifiers have not yet been well characterized.

## HISTONE MODIFICATION IN DROUGHT STRESS RESPONSE

**FIGURE 1:**
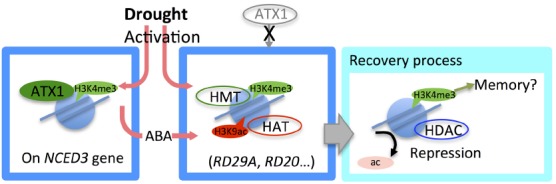
**Epigenetic regulation in the drought stress response.** Under drought stress, *NCED3*, a key gene in the abscisic acid (ABA) biosynthesis pathway, is activated with an increase in H3K4me3 marked by the histone methyltransferase ATX1. Active histone marks, such as H3K4me3 and H3K9ac were enriched on many drought-responsive genes. During recovery from drought stress, H3K9ac is rapidly removed, while H3K4me3 is removed more slowly, indicating that H3K4me3 may be involved in epigenetic memory.

Drought stress affects plant growth and survival. Gene regulatory networks associated with the drought stress response in plants have been studied by analyzing drought stress-responsive genes that encode functional and regulatory proteins, such as transcription factors (Shinozaki and Yamaguchi-Shinozaki, 2007; Fujita et al., 2011; Osakabe et al., 2014). The expression of drought stress-responsive genes is positively correlated with the intensity of drought stress (Matsui et al., 2008). The transcriptional responsiveness of drought stress-upregulated genes was found to be correlated with changes in histone modification and nucleosome density (Kim et al., 2008, 2012; To and Kim, 2014). Under strong drought conditions, the histone modifications H3K4me3 and H3K9ac on drought stress-upregulated genes, such as *RD20* and *RD29A*, were more highly enriched than under moderate drought conditions (Kim et al., 2008, 2012; Figure 1). Furthermore, under moderate drought conditions, there was little nucleosome loss from the *RD29A* region (Kim et al., 2008), while under strong drought conditions, notable nucleosome loss occurred in the same gene region (Kim et al., 2012). These results indicated that epigenetic responsiveness depended on the intensity of the drought stress.

To regulate gene activity, an epigenetic mode shift is required for precise transcriptional regulation. To fully repress the expression of stress-upregulated genes and to reset chromatin status under non-drought conditions, aggressive histone deacetylation and nucleosome replacement are required (Kim et al., 2012). During recovery from drought stress, H3K9ac rapidly decreased on the drought stress-upregulated genes, *RD29A*, *RD20*, and *AtGOLS2* (Figure 1). Simultaneously, RNA polymerase II was shown to be rapidly removed from those gene regions. These results suggested that histone deacetylation was promoted to remove the H3K9ac in conjunction with transcriptional repression (Figure 1). H3K4me3 was removed more slowly than H3K9ac; its enrichment decreased gradually with rehydration treatment (Figure 1). Surprisingly, the results of our previous study suggested that nucleosome density on drought stress-inducible genes behaved in an oscillation-like manner during recovery from dehydration (Kim et al., 2012), suggesting that nucleosome replacement is required to remove H3K4me3 and to reset the chromatin status.

It has been reported that drought stress response is memorized via histone modification on several drought stress-upregulated genes (Ding et al., 2012). H3K4me3 is a good marker of gene activation and stress memory (Oh et al., 2008; Zhang et al., 2009; Ding et al., 2011, 2012; Kim et al., 2012; Figure 1). H3K4me3 enrichment was found to be correlated with transcriptionally active gene regions based on a genome-wide analysis using ChIP-seq (van Dijk et al., 2010). Interestingly, in *Arabidopsis*, genome-wide H3K4me3 enrichment peaked 300 bp downstream from the transcriptional start site, similar to the H3K4me3 distribution pattern found in human cells (Barski et al., 2007). In contrast, H3K4me3 was distributed broadly on many dehydration/ABA (abscisic acid) inducible genes (van Dijk et al., 2010).

H3K4me3 modification by the HMT *Arabidopsis* trithorax-like 1 (ATX1) is involved in the activation of *NCED3*, which encodes a key enzyme in the ABA biosynthesis pathway, under drought stress conditions (Ding et al., 2011; Figure 1). ATX1 binding to the *NCED3* region increased under drought treatment. The *atx1* mutant plant had remarkably lower enrichments of RNA pol II and H3K4me3 under drought stress conditions compared with the wild type. Transcript levels of several drought stress and ABA-upregulated genes, such as *RD29A* and *RD29B*, were reduced during drought treatment in the *atx1* mutant. By training with multiple drought treatments, the enriched H3K4me3 levels in the *RD29B* and *RAB18* gene regions were maintained after rehydration, and Ser5P RNA pol II, the activated form of RNA pol II, was found to be stalled on these trained gene regions (Ding et al., 2012). The H3K4me3 level on *RD29B* was lower in the *atx1* mutant than in the wild-type plant after drought stress (Ding et al., 2012). Thus, ATX1-modified H3K4me3 may be considered to have an important role in regulation of the drought-responsive gene network via *NCED3*; however, ATX1 does not seem to have a critical impact on drought stress-memory in *Arabidopsis*.

Histone acetylation status has also been correlated with drought stress and ABA responses in plants. For example, in response to drought stress, the histone acetylation level increased on the drought-responsive genes, such as *RD20*, *RD29A*, and *RD29B* (Kim et al., 2008) and H3K9ac was enriched rapidly in these gene regions. Interestingly, specific patterns of histone acetylation were found on each drought-responsive gene, suggesting that differences in histone modification may contribute to the responsiveness of these genes.

In rice, drought stress significantly induced four HAT genes (*OsHAC703*, *OsHAG703*, *OsHAF701*, and *OsHAM701*; Fang et al., 2014) and enhanced acetylation of H3K9, H3K18, H3K27, and H4K5 under drought stress conditions was found by western blotting analysis. In barley (*Hordeum vulgare* L.), the expression of all three GNAT-MYST family HAT genes (*HvMYST*, *HvELP3*, and *HvGCN5*) was induced by ABA treatment (Papaefthimiou et al., 2010). Phylogenetically, *OsHAM701* and *OsHAG703* belong to the *HvMYST* and *HvELP3* clades, respectively. Thus, the epigenetic response to drought stress may be partially conserved between rice and barley.

The HD2-type HDACs, HD2A (HDT1), HD2B (HDT2), HD2C (HDT3), and HD2D (HDT4) belong to a plant-specific HDAC family in *Arabidopsis*, rice, barley, and tomato (Dangl et al., 2001; Pandey et al., 2002; Wu et al., 2003; Demetriou et al., 2009). *AtHD2C*-overexpressing *Arabidopsis* plants showed ABA insensitivity, reduced transpiration, and enhanced tolerance to drought and salt stresses (Sridha and Wu, 2006). In barley, the expression of *HD2* genes responded to stress-related plant hormones such as ABA, jasmonic acid (JA), and salicylic acid (SA; Demetriou et al., 2009). These results suggested that *HD2* genes play roles in resistance to abiotic and biotic stresses in monocot and dicot plants.

## HISTONE MODIFICATION IN SALT STRESS RESPONSE

Understanding the involvement of histone modifications in salt stress responses has gradually progressed and three histone modifications, acetylation, methylation, and phosphorylation, have been shown to influence the salinity stress response in plants.

Histone acetylation is generally correlated with gene activation, and is controlled by antagonistic actions between the *HAT* and *HDAC* proteins. In maize roots, the upregulation of cell wall-related genes, such as *ZmEXPB2* and *ZmXET1*, has been associated with increased H3K9 acetylation in the promoter and coding regions, which is thought to be necessary for high salinity response. It has been speculated that the upregulation of these genes might be mediated by two *HAT* genes (*ZmHATB* and *ZmGCN5*), because their mRNA expression was found to increase under salt stress conditions (Li et al., 2014). In *Arabidopsis*, a mutant for the transcriptional adaptor ADA2b, which modulates HAT activity, showed hypersensitivity to salt (Kaldis et al., 2011), suggesting that HATs play a pivotal role in salinity tolerance. However, *Arabidopsis* mutants for HDAC proteins such as HD2C, histone deacetylase 6 (HDA6), and histone deacetylase 19 (HDA19), which should increase histone acetylation, showed hypersensitivity to salt (Chen and Wu, 2010; Chen et al., 2010a; Luo et al., 2012b).

**FIGURE 2:**
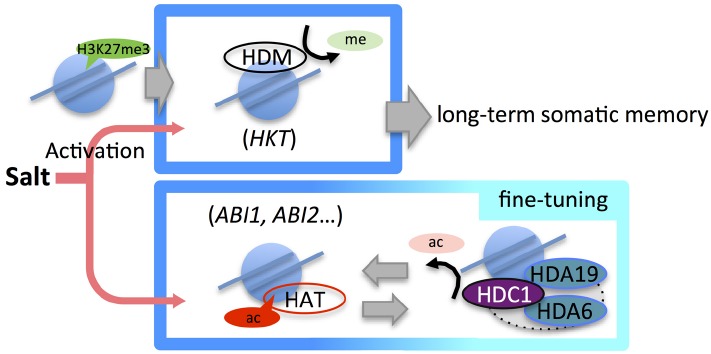
**Epigenetic regulation in the high-salinity stress response.** Salt stress induces the activation of high-salinity stress-responsive genes with changes in several histone modification marks. Treatment with low concentrations of salt prior to a second stress (priming) induces reduction of H3K27me3 in *HKT*, which controls Na^+^ partitioning. Priming-induced low H3K27me3 levels in *HKT* established long-term memory for rapid response to a second stress (top). Under salt stress, the expression of genes such as *ABI1* and *ABI2*, which are associated with the abscisic acid (ABA) signaling pathway, is fine-tuned by epigenetic regulation mechanisms involving histone acetylation (bottom).

In *Arabidopsis*, a HDAC complex, which included histone deacetylase complex1 (HDC1), HDA6, HDA19, and *Arabidopsis* Swi-Indipendent3 (AtSin3) was reported (Perrella et al., 2013). HDACs often form functional complexes of multiple proteins to regulate gene activity in eukaryotes (Carrozza et al., 2005a,b; Roguev and Krogan, 2007; Yang and Seto, 2008; Chen et al., 2012). HDA6, HDA19, and AtSin3 are considered as homologs of yeast (*Saccharomyces cerevisiae*) ScRPD3 deacetylase and ScSin3 proteins. Likewise, HDC1 was found to be an *Arabidopsis* homolog of ScRXT3. Interestingly, it was reported that the yeast RPD3L complex, comprising ScRPD3, ScSin3, and ScRXT3, responded to heat stress in yeast (Ruiz-Roig et al., 2010). Moreover, the yeast RPD3μ complex that consists of ScRPD3, ScSNT2, and ScECM5 may mediate an oxidative stress response in yeast (McDaniel and Strahl, 2013). These reports of yeast RPD3 complexes indicate that the *Arabidopsis* HDAC complex involving HDC1, HDA6, and HDA19 may function in the plant’s abiotic stress responses. Indeed, an *hdc1* mutant of *Arabidopsis* induced the expression of *ABA1*, *ABA3*, and *RAB18*, while overexpression of HDC1 led to repressed expression of *ABA1*, *RAB18*, and *RD29A* under high-salinity conditions (Perrella et al., 2013). These results indicated that histone modifier complexes involving *HATs* and HDACs might be required to fine-tune the histone acetylation status for plant adaptation in response to high-salinity stress (Figure 2, top).

Most reports into the salt stress response in plants have addressed how changes in histone acetylation states are connected to changes in gene expression. Indeed, it has often been the case that histone methylation was surveyed to confirm the interaction with acetylation in the stress response. However, an interesting study by Sani et al. (2013) aimed to decipher the epigenetic action of long-term somatic memory for salinity response. They showed that Na^+^-pretreated plants showed more drought tolerance than non-treated control plants after a Na^+^ stress-free period. A change in the H3K27me3 level around the *HKT1* gene, which encodes a high-affinity K(^+^) transporter, induced by mild salt stress was found to be a candidate for explaining the physiological effects caused by the priming treatment (Sani et al., 2013). Many studies have contributed to the current understanding of the core salt-tolerance mechanisms in plants (Deinlein et al., 2014 and references therein). In plant salinity tolerance, maintaining low Na^+^ levels is important to mitigate ionic Na^+^ stress. The sodium transporter HKT1 controls root–shoot Na^+^ partitioning and plays a major role in salt tolerance (Apse et al., 1999; Horie et al., 2009). Sani et al. (2013) identified the shortening and fractionation of H3K27me3 islands after priming treatment by whole-genome ChIP-seq, but found that H3K4me2, H3K4me3, and H3K9me2 islands rarely changed. In the chromatin transition, a long-lasting loss of H3K27me3 was found to occur, implying a release from gene repression in the island and resulting in a rapid and transient increase in the *HKT1* mRNA level (Figure 2, bottom; Sani et al., 2013). It is still unclear which genes contribute to enhanced drought stress tolerance in response to mild salt stress priming; however, Sani et al. (2013) have reported that the shortening and fractionation of H3K27me3 in a large number of genes including HKT1 play an important role in somatic memory caused by salt response.

In tobacco BY-2 and *Arabidopsis* T87 culture cells, rapid transient upregulation of histone H3 Ser-10 phosphorylation occurs, and H3 phosphoacetylation and histone H4 acetylation follow immediately. Interestingly, the onset and persistence of these H3 histone modifications differed between cold and high salinity stress responses (Sokol et al., 2007), suggesting that histone phosphorylation along with other histone modification might be controlled selectively by each stress type, although H3 Ser-10 phosphorylation by itself occurred on a massive scale during active cell division (Paulson and Taylor, 1982).

## HISTONE MODIFICATION IN HEAT STRESS RESPONSE

**FIGURE 3:**
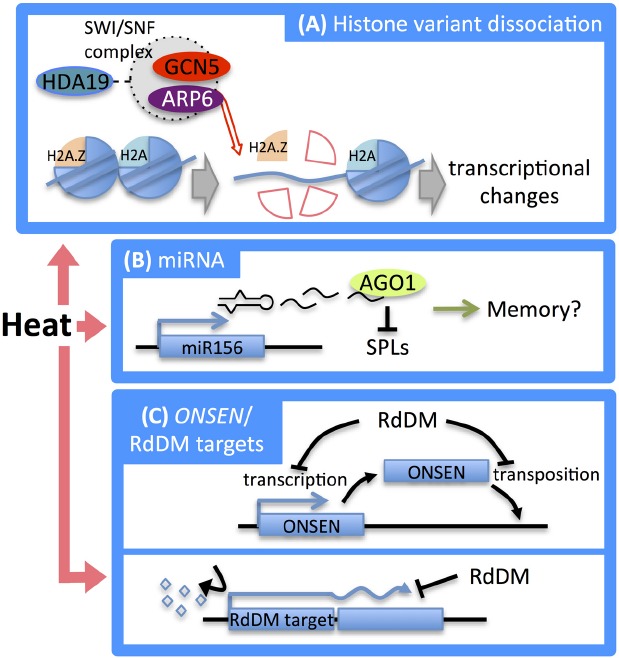
**Epigenetic regulation in the heat stress response. (A)** At high temperatures, H2A.Z is dissociated by the SWI/SNF chromatin remodeling complex which contains ARP6. This dissociation causes transcriptional changes in downstream genes. **(B)** After heat stress treatment, the expression of the microRNA miR156, which targets *SPL* genes, is increased. *ago1* mutants of *Arabidopsis* show defects in heat acclimation, implying involvement of miR156-mediated post-transcriptional regulation of *SPL* genes in heat stress memory. **(C)** Heat stress induced activation of the *ONSEN* retrotransposon. RdDM suppresses transcriptional and transpositional activation of *ONSEN* (top). Heat stress-induced DNA methylation causes aberrant transcription of genes that are located proximate to RdDM targets (bottom). Induction of DNA methylation restores normal transcription (bottom).

Histone variant deposition and histone modifications through acetylation and/or SUMOylation are considered to be involved in the thermal stress response. SUMO (small ubiquitin-related modifier) was identified as a reversible post-translational modifier that plays an important role in the regulation of protein interactions in eukaryotes. Recent studies have revealed that the occupancy of each histone variant of a core histone, in particular H2A and H3, plays important roles in not only gene expression, but also in the repair of DNA breaks and the assembly of chromosome centromeres in eukaryotes (Mizuguchi et al., 2004; Lu et al., 2009; Choi et al., 2013). In *Arabidopsis*, it has been suggested that H2A.Z deposition in gene bodies promotes variability in the levels and patterns of gene expression (Zilberman et al., 2008; Lu et al., 2009; Coleman-Derr and Zilberman, 2012). In addition to the regulation of gene expression, a genetic screen revealed that H2A.Z contributed to the thermosensory response via its nucleosome occupancy. A screen of *Arabidopsis* mutants deficient in temperature sensing under ambient temperatures (12–27°C) identified *ARP6* (actin-related protein 6) as a regulator of the coordinated changes in gene expression in response to ambient temperature changes (Kumar and Wigge, 2010). *ARP6* encodes a subunit of the SWR1 complex (March-Díaz and Reyes, 2009) that is necessary for inserting the alternative histone H2A.Z into nucleosomes replacing the core histone H2A, and could be involved in temperature sensing (Deal and Henikoff, 2010; Kumar and Wigge, 2010). In *Brachypodium*, the impairment of H2A.Z deposition reduced grain yield under heat stress conditions (Boden et al., 2013). Together, these studies suggest that H2A.Z deposition plays an important role in thermal stress responses. However, other modification such as H3K56 acetylation facilitated by histone chaperon AtASF1A/B is associated with nucleosome loss and causes the accumulation of RNA polymerase II (Weng et al., 2014), and the activation of transcription factors (Sidaway-Lee et al., 2014) also operate in response to heat stress. Because histone acetylation was shown to be necessary for H2A.Z deposition in yeast (Watanabe et al., 2013), histone modification may control the deposition of H2A.Z and possibly other histone variants in the heat stress response in plants. Further analyses are needed to uncover what kinds of histone modification and histone deposition contribute to the heat stress response in plants (Figure 3A).

In animals, SUMOylation is an important regulatory mechanism for the control of transcriptional repression mediated by histone modifiers such as HDACs (Kirsh et al., 2002; Geiss-Friedlander and Melchior, 2007). In *Arabidopsis*, the understanding of SUMOylation under heat stress conditions has progressed and a variety of chromatin modifier and components such as H2B, GCN5, HDA19, and the deubiquitinating enzyme UBP26, which removes ubiquinone bound to H2B, have been found to be SUMOylated (Miller et al., 2010). Interestingly, heat stress treatment (37°C for 30 min) was reported to decrease the SUMOylation status of H2B and increase the status of the GCN5 HAT (Miller et al., 2010, 2013).

There is growing evidence that epigenetic mechanisms contribute to stress memory in plants. Epigenetic memory for stress responses has been summarized in excellent reviews (Chinnusamy and Zhu, 2009; Mirouze and Paszkowski, 2011). A recent study indicated that the microRNA miR156 is involved in memory of heat stress (Stief et al., 2014). *Arabidopsis* plants that are pre-treated with moderate heat stress (priming) were found to acquire thermotolerance (heat stress memory) to severe heat stress. Hypomorphic mutants of *AGO1*, a component required for microRNA-mediated post-transcriptional gene silencing, were defective in maintaining the acquired thermotolerance. During priming heat stress, the amount of miR156 increased, while the SPL (squamosal-promoter binding-like) transcription factor genes, which are master regulators of developmental transitions and targeted by miR156, were downregulated. An acquired adaptation to recurring heat stress was reported to be achieved by miR156-mediated post-transcriptional regulation of *SPL* genes (Stief et al., 2014; Figure 3B).

## HISTONE MODIFICATION IN COLD STRESS RESPONSE

In *Arabidopsis*, vernalization, which is the best characterized pathway involved in epigenetic regulation induced by environmental stresses, can be achieved by long-term exposure to cold temperatures (Song et al., 2012a). Short term exposure to non-freezing low temperatures enhances freezing tolerance, and this process is known as cold acclimation. Both of these processes are low temperature responses; however, they are mediated by independent pathways (Bond et al., 2011).

**FIGURE 4:**
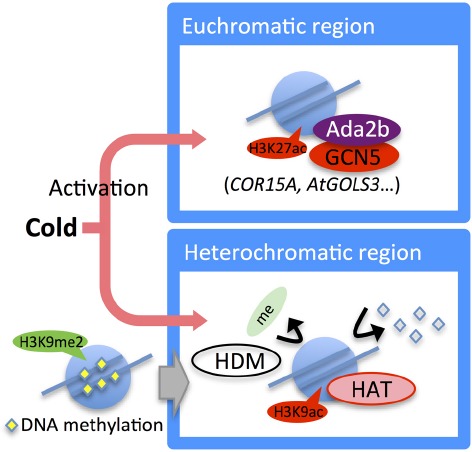
**Epigenetic regulation in the cold stress response.** Expression of the *HDA6* gene is induced by long-term cold treatment. Under cold stress, the histone acetylation level in cold-responsive genes (e.g., *COR15A* and *AtGolS3*) is increased by Ada2b and GCN5. In heterochromatic regions, H3K9me2 is removed and converted to acetylation mark. DNA methylation also decreased.

Low temperature treatment induces genome-wide transcriptional changes. In *Arabidopsis*, it has been estimated that the transcription of 3 to 20% of the genes change in response to cold stress (Chinnusamy et al., 2007; Matsui et al., 2008). Some epigenetic regulators are transcriptionally upregulated under cold stress conditions, suggesting that their upregulation may cause epigenetic and transcriptional changes of the target genes. In *Arabidopsis*, the expression of HDA6 was induced by long-term low temperature treatments (Figure 4), and a mutation in this gene resulted in sensitivity to freezing stress (To et al., 2011b). In maize, the expression of HDACs was upregulated during cold acclimation, and global deacetylation at H3 and H4 was observed (Hu et al., 2011). Moreover, during cold stress treatment, heterochromatic tandem repeats were selectively unsilenced, then H3K9ac was accumulated and DNA methylation and H3K9me2 were reduced in the unsilenced regions (Hu et al., 2012; Figure 4).

Local epigenetic changes in cold-responsive genes were also induced. In *Arabidopsis*, H3K27me3 on the cold-responsive genes, *COR15A*, and *AtGolS3*, decreased gradually in both a histone occupancy-dependent and -independent manner during cold stress treatment (Kwon et al., 2009). In maize, histone acetylation in cold-responsive genes such as *ZmDREB1* and *ZmCOR413* increased (Hu et al., 2011). Histone acetylation of *OsDREB1b* was induced by cold stress in rice (Roy et al., 2014). Thus, epigenetic changes induced by cold stress are likely to contribute to the acquirement of stress tolerance through changes in the expression of cold-responsive genes (Figure 4).

Several epigenetic regulators that are involved in the cold stress response were characterized by forward and reverse genetic studies. ADA2b, a transcriptional activator of HATs (Stockinger et al., 2001), was reported to interact with GCN5, an *Arabidopsis* HAT, and enhance the HAT activity of GCN5. In *ada2b* and *gcn5* mutants of *Arabidopsis*, the induction of COR (cold-regulated) transcripts by low temperature was delayed, and the final mRNA expression levels were reduced. The *ada2b* mutants showed increased freezing tolerance, indicating that ADA2b may function in the repression of freezing tolerance through histone acetylation (Vlachonasios et al., 2003).

Under cold stress, HOS1 (high expression of osmotically responsive gene 1), a RING finger E3 ligase, is a negative regulator of cold-responsive genes such as *CBFs/DREB1s* and *COR/RD/KIN*, and the ICE1 transcription factor (Dong et al., 2006). A recent study showed that HOS1 regulated chromatin status in the *FLC* locus by dissociating HDA6 from *FLC* chromatin (Jung et al., 2013).

The expression of HOS15, a WD40-repeat protein, was found to be induced by abiotic stresses including cold stress (Zhu et al., 2008). In a *hos15* mutant of *Arabidopsis*, transcripts of stress-regulated genes, such as *RD29A*, were reported to accumulate. Moreover, *hos15* mutants showed hypersensitivity to freezing stress. HOS15 shares sequence similarity with human TBL1 (transducin-beta like protein 1), which is a component of the SMRT/N-CoR repressor complex that associates with HDACs. It has been reported that HOS15 is involved in cold tolerance through the regulation of deacetylation at histone H4 (Zhu et al., 2008).

Compared with vernalization, the understanding of epigenetic regulation underlying the cold acclimation mechanism is limited. Further research is needed to increase the knowledge about this process.

## REGULATION MECHANISM OF DNA METHYLATION IN PLANTS

DNA methylation is another conserved epigenetic modification in eukaryotic organisms. Although the cytosines in CG sites are methylated in differentiated mammalian cells, cytosines in all contexts (CG, CHG, and CHH, where H indicates A, T, or C) can be methylated in plants. While DNA methylation is considered to be a recessive mark, recent studies have revealed that CG methylation can occur within the coding regions of transcribed genes (gene body methylation). Non-CG methylation is found exclusively in heterochromatin. DNA methylation status is regulated by *de novo* methylation, maintenance methylation, and active demethylation.

In plants, *de novo* DNA methylation was shown to be induced by a 24-nt short interfering (si) RNA-mediated pathway called RNA-directed DNA methylation (RdDM; Matzke and Mosher, 2014). In RdDM, the plant-specific RNA polymerases Pol IV and Pol V have important functions. These polymerases are evolutionarily related to Pol II but have different roles in RdDM, namely siRNA biogenesis (Pol IV) and recruitment of the silencing complex (Pol V). In brief, 24-nt siRNA is generated from Pol IV transcripts through processes mediated by RNA interference (RNAi) machineries such as RDR2 (RNA-dependent RNA polymerase 2) and DCL3 (Dicer-like 3), Then, the siRNA is incorporated into AGO4 (Argonaute 4). Chromatin association of Pol IV partially depends on SHH1 (sawadee homeodomain homolog 1), which can recognize unmethylated K4 and methylated K9 of histone H3 (Law et al., 2013), while Pol V transcribe genes from target loci and the siRNA-AGO4 complex is recruited to the transcript in a sequence-dependent manner. For transcription by Pol V, the so-called DDR complex, which is composed of DRD1 (defective in RNA-directed DNA methylation), DMS3 (defective in meristem silencing 3), and RDM1 (RNA-directed DNA methylation 1), is required (Kanno et al., 2004, 2008; Gao et al., 2010; Law et al., 2010). Finally, Dnmt3 class *de novo* methylase DRM2 (domains rearranged methylase 2) is recruited to target loci where it adds a methyl group to cytosines (Cao and Jacobsen, 2002). Several other effectors required for RdDM are discussed in a recent review by Matzke and Mosher (2014).

Induced DNA methylation is retained by maintenance methylases. CG methylation is maintained by conserved Dnmt1 class methylase MET1 (methyltransferase 1), while the plant-specific methylase CMT3 (chromomethylase 3) is required for maintenance of non-CG methylation, especially CHG methylation. Essential to this process is histone H3K9 dimethylation (H3K9me2), which is mediated by KYP/SUVH4, and CMT3 and KYP form self-reinforcement mechanism between DNA methylation and histone methylation (Jackson et al., 2002; Johnson et al., 2007). Maintenance of CHH methylation is regulated by both RdDM and the recently characterized CMT2. In addition to these DNA methyltransferases, chromatin remodeling factor DDM1 (decrease in DNA methylation 1) is also required (Vongs et al., 1993; Jeddeloh et al., 1999; Zemach et al., 2013). DDM1 is necessary to recruit maintenance methylases, such as CMT2, CMT3, and MET1 to linker histone H1 binding regions (Zemach et al., 2013).

DNA methylation is reversible. Active DNA demethylation was found to be achieved through a base excision–repair pathway (Zhu, 2009). The *Arabidopsis* genome encodes four DNA demethylase genes. *DME* (demeter) was shown to be preferentially expressed in central cells of female gametophytes, where it induces maternal allele-specific DNA demethylation of imprinted genes such as *MEA* (medea) and *FWA* (flowering Wageningen; Choi et al., 2002; Kinoshita et al., 2004). Recent studies revealed that *DME* was also expressed in the vegetative cells of pollen, but not in sperm cells, and that it played important roles in the establishment of epigenetic status in gametophytes (Schoft et al., 2011; Ibarra et al., 2012). Other DNA demethylases, ROS1 (repressor of silencing 1), DML2 (demeter-like 2), and DML3, are expressed in a wide range of organs (Penterman et al., 2007). Forward and reverse genetic screening revealed other factors that were required for active demethylation; for example, ROS3, an RNA binding protein, ZDP (zinc finger DNA 3′ phosphoesterase), a DNA 3′ phosphatase, and a HAT IDM1 (increase in DNA methylation 1) that can bind with methylated DNA and unmethylated histone H3K4 (Zheng et al., 2009; Martínez-Macías et al., 2012; Qian et al., 2012).

Although interaction between DNA methylation and histone modification, especially between non-CG methylation and H3K9me2, has been implicated, the current understanding of DNA methylation regulation in response to stress is limited compared to the better understanding of histone regulation. Further research will help clarify the underlying mechanisms that connect histone modification, DNA methylation, and stress responses.

## DNA METHYLATION IN ABIOTIC STRESS RESPONSE

The involvement of DNA methylation in the stress response is still poorly understood; however, recent studies have implicated the involvement of this epigenetic modification in the stress response. Global changes in DNA methylation, including hyper- and hypo-methylation, in response to abiotic stress have been reported in several plant species (Boyko et al., 2010; Bilichak et al., 2012; Karan et al., 2012; Wang et al., 2014) and alterations of DNA methylation in some stress-responsive genes have also been reported. Oxidative stress induced demethylation and transcriptional activation of *NtGPDL* (glycerophosphodiesterase-like protein) in *Nicotiana tabacum* (Choi and Sano, 2007). In soybean, salinity stress treatment induced the reduction of DNA methylation and transcriptional activation in genes that encoded salt stress-responsive transcription factors (Song et al., 2012b). Induction of DNA methylation by abiotic stress treatments has also been reported. In a low relative humidity stress condition, the numbers of stomata on the leaf epidermis were found to decrease in *Arabidopsis*. DNA methylation and transcriptional suppression in two positive regulator genes for stomatal development, *SPCH* (speechless) and *FAMA*, were reported to be induced by low relative humidity stress in *Arabidopsis* (Tricker et al., 2012). Thus, DNA methylation may play an important role in the transcriptional regulation of stress-responsive genes.

The system that regulates DNA methylation also functions as a genomic defense mechanism against abiotic stresses. The siRNA-mediated RdDM pathway restricts transcriptional activity and retrotransposition of the *copia*-type retrotransposon *ONSEN* triggered by heat stress (Ito et al., 2011; Figure 3C, top). *ONSEN* is transcriptionally reactivated by heat treatment, and during the recovery period after heat treatment the number of transcripts was found to diminish gradually. Although ONSEN is reactivated by heat stress, transposition has not been observed in wild-type plants. Interestingly, transcriptional reactivation was enhanced and transposition occurred in *Arabidopsis* mutants of RdDM components such as *nrpd1* (largest subunit of Pol IV), *nrpd2* (common second largest subunit of Pol IV and Pol V), and *rdr2* (Ito et al., 2011), indicating that the RdDM pathway restricts transcriptional reactivation and retrotransposition of *ONSEN* induced by heat treatment (Figure 3C, top).

RNA-directed DNA methylation is required for basal tolerance against heat stress (Popova et al., 2013). The *nrpd2* mutants showed hypersensitivity against heat stress and aberrant read-through transcription induced by heat treatment was diminished during the recovery process after heat stress in wild-type plants, but not in *nrpd2* mutants (Popova et al., 2013). Such misregulated genes harbor RdDM target sequences, such as transposon remnants, in proximal regions. Heat treatment was found to remove CHH methylation at the transposon remnants in wild-type plants and to induce aberrant transcription of RdDM targets and their nearby genes (Popova et al., 2013). Induction of CHH demethylation on RdDM targets during the recovery period diminished such aberrant transcription in wild-type plants; however, a hypomethylated status continued and misregulation of gene expression was not restored in *nrpd2* mutants. These results indicated that the altered heat responsiveness in *nrpd2* mutants may be caused by defective epigenetic regulation of nearby RdDM targets (Popova et al., 2013; Figure 3C,bottom).

## CONCLUSIONS AND PERSPECTIVES

It is currently understood that the regulation of abiotic stress-responsive genes is related to chromatin alterations. This new understanding has introduced new research aspects to the study of plant abiotic stress responses. It has been shown that dynamic epigenetic changes occur in response to abiotic stresses, and the epigenetic response and memory of gene activation in response to abiotic stresses have become major topics of interest. However, the entire correlation network between abiotic stress responses and epigenetic information, such as the stress-responsive epigenetic modifiers, the targeted stress-responsive genes, and the specific histone modification sites, remain unclear. Investigating the direct effects of histone modification in plants is difficult, because plant genomes harbor multiple copies of histone genes. ChIP assays have proven valuable in helping to identify the histone modifications responsible for epigenetic regulation; however, the results obtained by these assays provide only direct/indirect candidate residues that may be targets for modification. Identifying the functions of histone modification in plant epigenetic studies directly is still a challenge. Generation of mutants of the histone modifiers and of mutated amino acid residues in the histone N-tail by CRISPR/Cas system (Cong et al., 2013) will be an important contribution to these studies, because they will help to determine the superiority between transcriptional changes and epigenetic alterations. Moreover, there is a possibility that tissue-specific alterations are necessary for plants to acquire stress tolerance (Baxter et al., 2010); therefore, because it is difficult to detect tissue-specific alterations of histone modifications by ChIP experiments with the current resolution, the results of ChIP experiments should be evaluated cautiously. Reliable and higher resolution chromatin studies are undoubtedly required to reveal key epigenetic information involved in abiotic stress responses. In this review, we have shown that it is essential to establish experimental materials and efficient technologies for future intensive and acute analyses in plant epigenetics. When the master regulators of epigenetic regulation and novel epigenetic regulation mechanisms in the stress response are discovered, in-depth studies on plant abiotic stress responses will progress rapidly.

### Conflict of Interest Statement

The authors declare that the research was conducted in the absence of any commercial or financial relationships that could be construed as a potential conflict of interest.
